# Supplementary material for: Content-rich biological network constructed by mining PubMed abstracts
Source: BMC Bioinformatics. 2004 Oct 8;5:147. doi: 10.1186/1471-2105-5-147 (PMC528731; doi:10.1186/1471-2105-5-147)
Supplement: Additional File 2 — The original results of the above study (non-essential files are deleted to keep the file size under the limit set by BMC bioinformatics). [file 1471-2105-5-147-S2.bz2 › chilibotAdditionalFile2/dip20/326ID10638746E3625/html/TFRC_HFE.html]

 


 **TFRC** and **HFE** 
  
Found 145 abstracts in PubMed, retrieved 20.  
 

 What does Google say? 
 PDF only 
| .edu only 

---

**Interactive relationship** (e.g. stimulation, inhibition, etc)

**Inhibitory relationship**- Cells expressing a chimeric  **HFE**  protein with the cytoplasmic domain of the lysosomal protein, LAMP1, in place of its own  **HFE**  LAMP, show a decrease in the half life of the TfR  [ **TFRC** ]  implying that the interaction between  **HFE**  and TfR  [ **TFRC** ]  in endosomes targets the TfR  [ **TFRC** ]  to lysosomal compartments.  Ref: 12667138 Biochem J< MedlineTA>Biochem J,
- To test the effect of  **HFE**  on lowering intracellular iron levels independently of its association with TfR  [ **TFRC** ] , a mutated  **HFE**  fW81AHFE that shows greatly reduced affinity for the TfR  [ **TFRC** ]  was transfected into tetracycline controlled transactivator HeLa cells.  Ref: 12874382 Proc Natl Acad Sci U S AProc Natl Acad Sci U S A,
- However, cells expressing  **HFE**  still showed a decrease in Tf mediated iron uptake at concentrations of Tf sufficient to dissociate  **HFE**  from the TfR  [ **TFRC** ] .  Ref: 12874382 Proc Natl Acad Sci U S AProc Natl Acad Sci U S A,
**Neutral relationship**- At variance with  **TFRC** , interactions between TFR2 and  **HFE**  do not occur, at least in their soluble forms.  Ref: 12547237 Blood Cells Mol Dis< MedlineTA>Blood Cells Mol Dis,
- The interaction between TfR  [ **TFRC** ]  and  **HFE**  LAMP was confirmed by immunoprecipitation, in addition to immunofluorescence studies.  Ref: 12667138 Biochem J< MedlineTA>Biochem J,
- Recent studies have revealed that the wild type hemochromatosis  [ **HFE** ]  protein  **HFE**  interacts with the transferrin receptor  [ **TFRC** ]  TfR  [ **TFRC** ]  and modulates TfR  [ **TFRC** ]  mediated iron uptake by cells.  Ref: 12791823 J Nucl Med, 2003
- Recent reports suggest that the mutations of transferrin receptor  [ **TFRC** ]  2 TfR2 gene may be involved in non  **HFE**  hemochromatosis  [ **HFE** ] .  Ref: 12809944 Hepatol Res< MedlineTA>Hepatol Res, 2003
- Although the  **HFE**  protein appears to modulate the function of the transferrin receptor  [ **TFRC** ]  in vitro, its precise role in vivo remains obscure.  Ref: 12657433 Trends Mol Med, 2003
- This protein regulates transferrin receptor  [ **TFRC** ]  mediated iron uptake and mutations in its gene cause hereditary iron overload hemochromatosis  [ **HFE** ] .  Ref: 12461085 J Exp Med, 2002
- **HFE** , the protein mutated in Hereditary Hemochromatosis  [ **HFE** ]  Type 1, is known to interact with the transferrin receptor  [ **TFRC** ]  TfR  [ **TFRC** ]  on the cell surface and during endocytosis Gross, C.N. et al.  Ref: 12667138 Biochem J< MedlineTA>Biochem J, 2002
- Evidence for the interaction of the hereditary hemochromatosis protein  [ **HFE** ] ,  **HFE** , with transferrin receptor  [ **TFRC** ]  in endocytic compartments.  Ref: 12667138 Biochem J< MedlineTA>Biochem J, 2002
- CONCLUSION Our studies suggest that expression of wild type  **HFE**  in cells produces a decrease in 67 Ga uptake due to a reduction in available Tf binding sites for 67 Ga Tf on the TfR  [ **TFRC** ] .  Ref: 12791823 J Nucl Med, 2003
- Consistent with earlier observations in other cell lines, the expression of wild type or H63D, but not C282Y,  **HFE**  induces an apparent iron deficient phenotype, manifested in the activation of iron regulatory protein and concomitant increase in transferrin receptor  [ **TFRC** ]  levels and decrease in ferritin content.  Ref: 12464008 Biochem J, 2003

**Non-interactive relationship** (e.g. studied together, co-existance, homology, etc.)

- METHODS The expression and localization of  **HFE** , TfR1  [ **TFRC** ]  and TfR2 proteins in human platelets were examined using Western blotting and immunocytochemistry.  Ref: 12656741 Eur J Haematol, 2003
- Human platelets express hemochromatosis  [ **HFE** ]  protein  **HFE**  and transferrin receptor  [ **TFRC** ]  2.  Ref: 12656741 Eur J Haematol, 2003
- These results indicate that the association of  **HFE**  with TfR  [ **TFRC** ]  is not essential for its ability to lower intracellular iron stores.  Ref: 12874382 Proc Natl Acad Sci U S AProc Natl Acad Sci U S A, 2003
- Co localization of the mammalian hemochromatosis  [ **HFE** ]  gene product  **HFE**  and a newly identified transferrin receptor  [ **TFRC** ]  TfR2 in intestinal tissue and cells.  Ref: 12704209 J Histochem Cytochem, 2003
- The following candidate genes have been examined in detail to determine whether polymorphisms in them may be responsible for this variation transferrin, transferrin receptor  [ **TFRC** ]  1, transferrin receptor  [ **TFRC** ]  2, ferritin L, ferritin H, IRP1, IRP2,  **HFE** , beta 2 microglobulin, mobilferrin calreticulin, ceruloplasmin, ferroportin, NRAMP1, NRAMP2 DMT1, haptoglobin, heme oxygenase 1, heme oxygenase 2, hepcidin, USF2, ZIRTL, duodenal cytochrome b ferric reductase DCYTB, TNFalpha, keratin 8, and keratin 18.  Ref: 12547238 Blood Cells Mol Dis< MedlineTA>Blood Cells Mol Dis, 2003
- Coimmunoprecipitation studies using solubilized cell extracts demonstrated that transferrin Tf competed with  **HFE**  for binding to the transferrin receptor  [ **TFRC** ]  TfR  [ **TFRC** ]  similar to previous in vitro studies using soluble truncated forms of  **HFE**  and the TfR  [ **TFRC** ] .  Ref: 12874382 Proc Natl Acad Sci U S AProc Natl Acad Sci U S A, 2003
- **HFE** , the protein defective in hereditary hemochromatosis  [ **HFE** ] , and transferrin receptor  [ **TFRC** ]  2 TfR2 are two novel protein candidates that could be involved in mechanisms of iron transport across the platelet plasma membrane.  Ref: 12656741 Eur J Haematol, 2003
- Addition of transferrin Tf to  **HFE**  LAMP expressing cells competes with  **HFE**  for binding to the TfR  [ **TFRC** ]  thereby increasing the half life of TfR  [ **TFRC** ]  and confirming that the  **HFE**  LAMP TfR  [ **TFRC** ]  complex is reaching the cell surface prior to entering the endosomal vesicles and trafficking to the lysosome.  Ref: 12667138 Biochem J< MedlineTA>Biochem J, 2003
- In iron overload, TfR1  [ **TFRC** ]  expression in the duodenum differed according to the presence or absence of the C282Y mutation in the  **HFE**  gene, being increased in  **HFE**  related hemochromatosis  [ **HFE** ]  and similar to controls in non  **HFE**  related iron overload.  Ref: 12547240 Blood Cells Mol Dis< MedlineTA>Blood Cells Mol Dis, 2003
- At concentrations of Tf approaching those found in the blood, no differences in Tf binding to cells were detected, which is consistent with the lower binding constant of  **HFE**  for TfR  [ **TFRC** ]  versus Tf.  Ref: 12874382 Proc Natl Acad Sci U S AProc Natl Acad Sci U S A, 2003
- The results indicate that  **HFE**  can lower intracellular iron levels independently of its interaction with the TfR  [ **TFRC** ] .  Ref: 12874382 Proc Natl Acad Sci U S AProc Natl Acad Sci U S A, 2003
- Such changes are detected by transferrin receptor  [ **TFRC** ]  2 and the  **HFE**  transferrin receptor  [ **TFRC** ]  1 complex.  Ref: 12737947 Blood Cells Mol Dis, 2003
- Duodenal expression of  **HFE**  and TfR2 but not TfR1  [ **TFRC** ]  in wild type mice and humans was restricted to crypt cells, in which they co localized.  Ref: 12704209 J Histochem Cytochem, 2003
- These results raise the possibility that interaction of  **HFE**  and TfR  [ **TFRC** ]  in intracellular vesicles may play an important role in determining the function of  **HFE**  in iron homeostasis, which is still unknown.  Ref: 12667138 Biochem J< MedlineTA>Biochem J, 2003
- Hence, the availability of plasma transferrin bound iron and the expression and function of transferrin receptors TfR1  [ **TFRC** ] ,  **HFE**  and DMT1 should all contribute to the absorptive capacity of villus enterocytes.  Ref: 12547229 Blood Cells Mol Dis< MedlineTA>Blood Cells Mol Dis, 2003
- Can defects in transferrin receptor  [ **TFRC** ]  2 and hereditary hemochromatosis  [ **HFE** ]  genes account for iron overload in HbH disease ? < ArticleTitle>.  Ref: 12667993 Blood Cells Mol Dis< MedlineTA>Blood Cells Mol Dis, 2003
- Human transferrin receptor  [ **TFRC** ]  2 is 45% identical with transferrin receptor  [ **TFRC** ]  1 in the extracellular domain, but contains no iron responsive element in its mRNA and is apparently not regulated by intracellular iron concentration nor by interaction with  **HFE** .  Ref: 12531241 Int J Biochem Cell Biol, 2003
- The transferrin receptor  [ **TFRC** ]  2 TFR2 and hereditary hemochromatosis  [ **HFE** ]   **HFE**  genes were examined to see if inheritance of these gene defects may be a possible cause of iron overload in 45 HbH patients.  Ref: 12667993 Blood Cells Mol Dis< MedlineTA>Blood Cells Mol Dis, 2003
- These aspects of the regulation and mechanism of iron absorption were investigated in genetically normal rats and mice, and in Belgrade anemic b b rats and  **HFE**  knockout mice.In most experiments the function of the TfR1  [ **TFRC** ]  was assessed by the uptake of radiolabeled transferrin bound iron given intravenously.  Ref: 12547229 Blood Cells Mol Dis< MedlineTA>Blood Cells Mol Dis, 2003
- Mutations in the  **HFE**  gene and a newly identified second transferrin receptor  [ **TFRC** ]  gene, TfR2, cause hemochromatosis  [ **HFE** ] .  Ref: 12704209 J Histochem Cytochem, 2003
- Hemochromatosis  [ **HFE** ]  due to mutations in transferrin receptor  [ **TFRC** ]  2.  Ref: 12547237 Blood Cells Mol Dis< MedlineTA>Blood Cells Mol Dis, 2003
- The presence of TfR2 and absence of TfR1  [ **TFRC** ]  suggests that  **HFE**  may serve a different function in platelets compared with the other  **HFE**  positive cell types, e.g. enterocytes, macrophages and syncytiotrophoblasts.  Ref: 12656741 Eur J Haematol, 2003
